# Singular Case of Asphyxia, Communicated for the “Examiner”

**Published:** 1849-10

**Authors:** Thomas F. Betton

**Affiliations:** Germantown, Pennsylvania


*Singular case of Asphyxia, communicated for the "Examiner," by* THOMAS F. BETTON, M. D., *Germantown, Pennsylvania.*

There occurred to me yesterday, a singular circumstance, the facts of which I send to you. It is curious, because uncommon.

A well digger, James D. Fenton, was employed by my neighbor Mr. Cox, of the Gen. Wayne Hotel, to do some repairs to his well. He was cautioned not to go down without the usual experiment of sending down a burning candle, which he did, and found that it was extinguished at about 10 feet from the surface of the water, say some 33 feet below the surface of the ground ; but he observed that it did not go entirely out, but that the coal on the wick still remained red.

Accordingly he descended, and feeling himself becoming faint, unhooked the rope from the bucket, and passed it round his body, and cried out to be hauled up The rope had a large iron hook affixed to the end. By some means or other the rope became disengaged, and slipped, either luckily or unluckily for him, around his neck. In this way he was drawn up suspended per collum, and with the iron hook pressing firmly under his right ear. To all appearances he was defunct. I was sent for, and living only a quarter of a mile off, saw him in five or ten minutes. I could barely perceive a faint pulsation of the heart. He was laid on a settee with his head raised on cushions. These I removed, dashed cold water freely in his face, and poured some brandy into his mouth. To my satisfaction he soon began to revive, his pulse returned, and in half an hour he was pretty well. He was totally unconscious of all that had passed, and even did not remember putting the rope about him. So far gone was he, that the sphincter ani was relaxed, to his great annoyance. He is well to-day.

*September* 8, 1849.

